# Galectin Family Members: Emerging Novel Targets for Lymphoma Therapy?

**DOI:** 10.3389/fonc.2022.889034

**Published:** 2022-05-23

**Authors:** Yuanwei Shi, Danting Tang, Xiaoqi Li, Xiaoli Xie, Yufu Ye, Lijuan Wang

**Affiliations:** ^1^School of Clinical Medicine, Weifang Medical University, Weifang, China; ^2^Central Laboratory, Linyi People’s Hospital, Linyi, China; ^3^Key Laboratory of Combined Multi-Organ Transplantation, Ministry of Public Health, First Affiliated Hospital, School of Medicine, Zhejiang University, Hangzhou, China; ^4^Department of Hepatobiliary and Pancreatic Surgery, First Affiliated Hospital, School of Medicine, Zhejiang University, Hangzhou, China; ^5^Linyi Key Laboratory of Tumor Biology, Linyi, China

**Keywords:** lymphoma, galectin-1, galectin-3, galectin-7, galectin-9

## Abstract

The galectin family of proteins has high affinity with β-galactoside-containing glycans. These proteins participate in cell growth and differentiation, cell adhesion, cell signal transduction, cell apoptosis, and other cellular activities. In recent years, a large number of studies have described the expression and correlation of galectins in different tumors. Each member of the family plays a vital role in tumor growth, progression, angiogenesis, adhesion, and tumor immune escape. Studies on the roles of galectins in lymphoma have mainly involved galectin-1, -3, -7, and -9. The results suggest that galectins may become novel targets for precise tumor treatment. This article reviews current research progress regarding galectins in lymphoma and provides new ideas for exploring them as novel targets for treating lymphoma and other important medical issues.

## Introduction

Lymphoma is the most common malignant tumor, originating from the lymphoid hematopoietic system (1). According to the latest global cancer statistics, the number of new cases of lymphoma worldwide in 2020 was 627,439, and the number of deaths was 283,169 (1). The main treatments for lymphoma are chemotherapy, radiotherapy, hematopoietic stem cell transplantation, molecular targeted therapy and immunotherapy (2, 3). A variety of emerging immunotherapeutic strategies, including monoclonal antibodies, antibody-drug conjugates, immunomodulatory drugs, immune checkpoint inhibitors, and CAR-T cell therapy, have been approved by the United States Food and Drug Administration for the treatment of lymphoma (3). However, improved therapies are needed.

Galectins belong to an endogenous lectin family and play important roles in cell differentiation, proliferation, apoptosis, adhesion, and migration (4). They have one or two carbohydrate recognition domains (CRDs) and high affinity for β-galactosides (5). To date, 16 members of the galectin family have been discovered and classified into three types: “proto-type“ galectins (including galectin-1, 2, 5, 7, 10, 11, 13, 14, 15,16) (6), “tandem-repeat” galectins (including galectin-4, 6, 8, 9, 12), and “chimera -type” galectin, galectin-3 (5). Galectins are widely expressed in various cells and recognize glycoconjugates containing β-galactosides on the cell surface, extracellular matrix, and intracellular vesicle cavities (4).

Galectins are expressed in various tumors; **Table 1** summarizes the functions and clinical significance of these proteins in different tumors. The galectin family also plays key roles in lymphoma by promoting tumor cell growth, survival, and tumor immune escape (88). Intervention with galectin inhibitors is emerging as an attractive treatment option for lymphoma (88). In subsequent sections, we summarize the latest research on galectins in lymphoma.

**Table 1:** The role of galectin in various tumors.

Galectin	Cancer type	Function and clinical significance	References
**Galectin-1**			
	Acute myelogenous leukemia	Differentiation, immunosuppression and chemotherapy resistance	(6)
	Acute lymphoblastic leukemia	Migration, anti-cytotoxic effect and tumor burden	(7)
	B-cell precursor acute lymphoblastic leukemia	Aggregation, adhesion, migration, survival, anti-chemotherapy-induced apoptosis and inhibition of macrophage-mediated cell killing	(8)
	Leukemic cutaneous T-cell lymphoma	Lower anti-tumor response and highly opportunistic infections	(9)
	Mixed lineage leukemia -rearranged B-lymphoblastic leukemias	Highly sensitive and specific reproducible marker	(10)
	Chronic myelogenous leukemia	Proliferation, apoptosis, differentiation, migration, resistance and long-term retention	(6, 11)
	Chronic lymphocytic leukemia	Anti-apoptosis, stimulation of cloning, activation of cancer cells, immunosuppression, progression and poor prognosis	(11–13)
	Anaplastic large cell lymphoma	Death sensitivity	(6)
	Classic Hodgkin’s lymphoma	Invasion, immune escape and diagnostic marker	(6)
	Hodgkin’s lymphoma	Predictive marker of disease progression	(12)
	Relapsed/Refractory lymphoma	Predictive biomarker	(6)
	Multiple myeloma	Bone marrow infiltration, proliferation, survival, angiogenesis	(6, 14)
	Head and neck tumors	Immune escape	(15)
	Oral squamous cell carcinoma	Migration and invasion	(16)
	Tongue squamous cell carcinoma	Metastasis, progression, clinical stage and progression	(17)
	Squamous cell carcinoma of the larynx and sublarynx	Rapid relapse and low survival rate, tumor development, prognosis and progression	(18)
	Gingival quamous cell carcinoma	Depth of invasion and lymph node metastasis	(19)
	Melanoma	Migration, angiogenesis and immune escape	(19)
	Thyroid cancer	Tumor cell proliferation	(20)
	Breast cancer	Angiogenesis, metastasis and infiltration	(20)
	Lung cancer	Migration, progression, angiogenesis, disease progression, chemotherapy resistance	(20)
	Liver cancer	Tumor cell growth, metastasis, invasion and cell adhesion, poor prognosis	(21)
	Stomach cancer	Proliferation, migration and angiogenesis	(20)
	Pancreatic cancer	Proliferation, invasion, angiogenesis, metastasis and immune escape	(20)
	Colorectal cancer	Proliferation, migration, invasion and progression	(20, 22)
	Cervical cancer	Migration and invasiveness	(22, 23)
	Ovarian cancer	Migration and invasiveness	(20)
	Endometrial cancer	Poor prognosis	(24)
	Bladder cancer	Disease progression	(25)
	Kidney cancer	Migration	(26)
	Prostate cancer	Migration, invasiveness and poor prognosis	(27)
	Neuroblastoma	Proliferation, migration and infiltration	(20)
**Galectin-2**			
	Breast cancer	Adhesion to vascular endothelium	(28)
	Stomach cancer	Metastasis	(29)
	Colorectal cancer	Adhesion to vascular endothelium	(28, 29)
	Bladder cancer	Tumor invasion	(25)
**Galectin-3**			
	Acute leukemia	chemotherapy resistance	(30)
	Acute myelogenous leukemia	Anti-apoptosis, adhesion, survival, proliferation, recurrence, independent poor prognostic factors and chemotherapy resistance	(7)
	Acute promyelocytic leukemia	High recurrence and mortality	(7)
	Chronic myelogenous leukemia	Proliferation, chemotherapy resistance and BM deposition	(31)
	B-cell precursor acute lymphoblastic leukemia	Migration, adhesion, chemotherapy resistance and inhibition of anti-leukemia response	(32)
	Chronic lymphocytic leukemia	Prognostic marker and effects on disease progression are contradictory	(33)
	Anaplastic large cell lymphoma	Biomarker	(34)
	Primary central nervous system lymphoma / Adult T-cell leukemia-lymphoma	Poor prognosis	(35, 36)
	Diffuse large B cell lymphoma	Metastasis, adhesion, anti-apoptosis and distinguishing from Follicular lymphoma	(6)
	Multiple myeloma	Metastasis, growth, migration, angiogenesis, adhesion, anti-apoptosis and chemotherapy resistance	(6, 14)
	Oral squamous cell carcinoma	Proliferation, migration and angiogenesis	(37)
	Tongue squamous cell carcinoma	Differentiation, metastasis and progression	(17)
	Melanoma	Prognosis and diagnosis	(37)
	Thyroid cancer	Angiogenesis Prognosis	(37, 38)
	Breast cancer	Metastasis, invasion, angiogenesis, recurrence and chemotherapy resistance	(37, 39)
	Lung cancer	Prognosis and recurrence	(40)
	Esophageal cancer	Angiogenesis, proliferation, migration and invasion	(38, 41)
	Liver cancer	Tissue differentiation, metastasis, invasion, progression and prognosis	(21)
	Stomach cancer	Metastasis, invasion and prognosis	(37, 42)
	Pancreatic cancer	Metastasis, invasion and prognosis	(37)
	Colorectal cancer	Proliferation, prognosis and chemotherapy resistance	(37, 42)
	Cervical cancer	Prognosis	(43)
	Ovarian cancer	Proliferation, migration, invasion, prognosis, chemotherapy resistance and survival	(44)
	Endometrial cancer	Migration	(45)
	Bladder cancer	Apoptosis, progression, invasion and chemotherapy resistance	(25)
	Kidney cancer	Classification and prognosis	(26)
	Prostate cancer	Migration, progression and early metastasis	(46)
	Neuroblastoma	Proliferation, migration and infiltration	(20)
**Galectin-4**			
	Tongue squamous cell carcinoma	Differentiation	(17)
	Lung cancer	Growth, invasion, tumor size and lymph node status	(47)
	Liver cancer	Growth, recurrence, metastasis, prognosis and survival	(48)
	Pancreatic cancer	Recurrence, prognosis, death	(49)
	Colorectal cancer	Growth and aggressiveness	(50, 51)
	Bladder cancer	Growth and metastasis	(52)
	Prostate cancer	Metastasis and progression	(53)
**Galectin-7**			
	Invasive mouse lymphoma model	Metastasis and invasion	(54, 55)
	Head and neck tumors	The degree of keratinization and differentiation	(56, 57)
	Oral squamous cell carcinoma	Malignancy, grade, migration and invasion	(58)
	Tongue squamous cell carcinoma	Relapse and prognosis	(17)
	Hypopharyngeal cancer	Progression	(59)
	Laryngeal cquamous cell carcinoma	Progression	(59)
	Melanoma	Apoptosis	(57)
	Thyroid cancer	Distinguish between benign and malignant	(57)
	Breast cancer	Metastasis, invasiveness, progression, chemotherapy resistance and apoptosis	(57)
	Esophageal cancer	Prognosis	(60)
	Stomach cancer	Growth and angiogenesis	(57)
	Colorectal cancer	Growth and angiogenesis	(57)
	Cervical cancer	Cell growth and angiogenesis	(61)
	Ovarian cancer	Proliferation, invasion, immunosuppression and prognosis	(57)
	Kidney cancer	Prognosis	(26, 62)
	Bladder cancer	Growth, angiogenesis and chemotherapy sensitivity	(57, 63)
	Prostate cancer	Cell growth and angiogenesis	(62)
	Neuroblastoma	Chemotherapy sensitivity	(64)
**Galectin-8**			
	Multiple myeloma	Adhesion and poor prognosis	(14)
	Head and neck cancer	Malignant transformation	(65)
	Thyroid cancer	Marker of Thyroid cancer	(66)
	Breast cancer	Cell adhesion,migration and tumorigenesis	(67)
	Lung cancer	Metastasis, cell adhesion and the degree of malignancy	(66, 67)
	Stomach cancer	Recurrence, survival and prognosis	(68)
	Colon cancer	Metastasis and growth cell adhesion	(67, 69)
	Cervical cancer	Cell adhesion	(67)
	Ovarian cancer	Prognosis	(70)
	Kidney cancer	Cancer cell necrosis and inflammation	(26)
	Bladder cancer	Grade and stage, relapse and prognosis	(71)
	Prostate cancer	Migration	(72)
	Neuroblastoma	Proliferation, migration and infiltration	(73)
**Galectin-9**			
	Acute myelogenous leukemia	Growth, progression, immunosuppression, impaired anti-tumor response, support for leukemia stem cells and poor prognosis	(6, 74, 75)
	Chronic myelogenous leukemia	Apoptosis	(76)
	Multidimensional scaling	Progress, low survival rate and poor prognosis	(7)
	Adult T-cell leukemia/ Adult T-Cell Leukemia-Lymphoma	Increases tumor burden and reflects immune-related adverse reactions of biological agents	(7, 77)
	Chronic lymphocytic leukemia	Proliferation, prognosis, immune escape	(7)
	Cutaneous T cell lymphoma	Lower anti-tumor response and highly opportunistic infections	(6)
	Multiple myeloma	Apoptosis, prognosis, growth inhibitory, anti-proliferation and anti-myeloma activity	(6, 78)
	Melanoma	Survival and chemotherapy sensitivity	(79)
	Breast cancer	Invasiveness, metastasis and survival	(80)
	Liver cancer	Cell adhesion, invasion, metastasis, apoptosis, immunosuppression, progression, prognosis and survival	(21)
	Esophageal cancer	Prognosis	(81)
	Stomach cancer	Survival	(82)
	Pancreatic cancer	Apoptosis, proliferation, growth and anti-tumor immunity	(83)
	Colon cancer	Proliferation	
	Cervical cancer	Differentiation and survival	(81)
	Ovarian cancer	Apoptosis	(84)
	Kidney cancer	Prognosis	(85)
	Bladder cancer	Prognosis	(79)
**Galectin-10**			
	Colorectal cancer	Survival	(86)
**Galectin-12**			
	Acute myelogenous leukemia	Prognosis	(33)
	Acute promyelocytic leukemia	Differentiation block	(87)

## Galectin-1

Galectin-1 has a molecular weight of 14.7 kDa and is encoded by the LGALS1 gene located at 22q12 (89). Galectin-1 exists and functions as a homodimer and is a typical cytoplasmic protein with an acetylated N-terminal (89). Galectin-1 is mainly expressed in the cytoplasm, shuttles between the cytoplasm and nucleus and is transferred to the cell membrane or extracellular matrix (89, 90). Galectin-1 has vital roles in tumorigenesis and tumor development. Overexpression of galectin-1 activates oncogenes, promotes the transformation of normal cells into malignant cells, and accelerates the growth and development of tumors by regulating the cell cycle (91). Galectin-1 promotes tumor migration, invasion, and angiogenesis through epithelial-mesenchymal transition (92), mediates the adhesion of tumor cells, and enhances the adhesion of cells to the extracellular matrix through glycoproteins in the basement membrane (93). Galectin-1 also accelerates the growth of tumor cells by promoting angiogenesis and the activation and proliferation of vascular endothelial cells (94).

Tumor cells weaken the function of immune cells by secreting galectin-1. This induces the tumor microenvironment to shift to the direction of immunosuppression and leads to immune escape of tumor cells (95). In addition, galectin-1 selectively reduces the viability of Th1 cells and participates in the immunosuppressive microenvironment by promoting the production of Th2 cytokines and the expansion of regulatory T cells (96).

**Figure 1:**
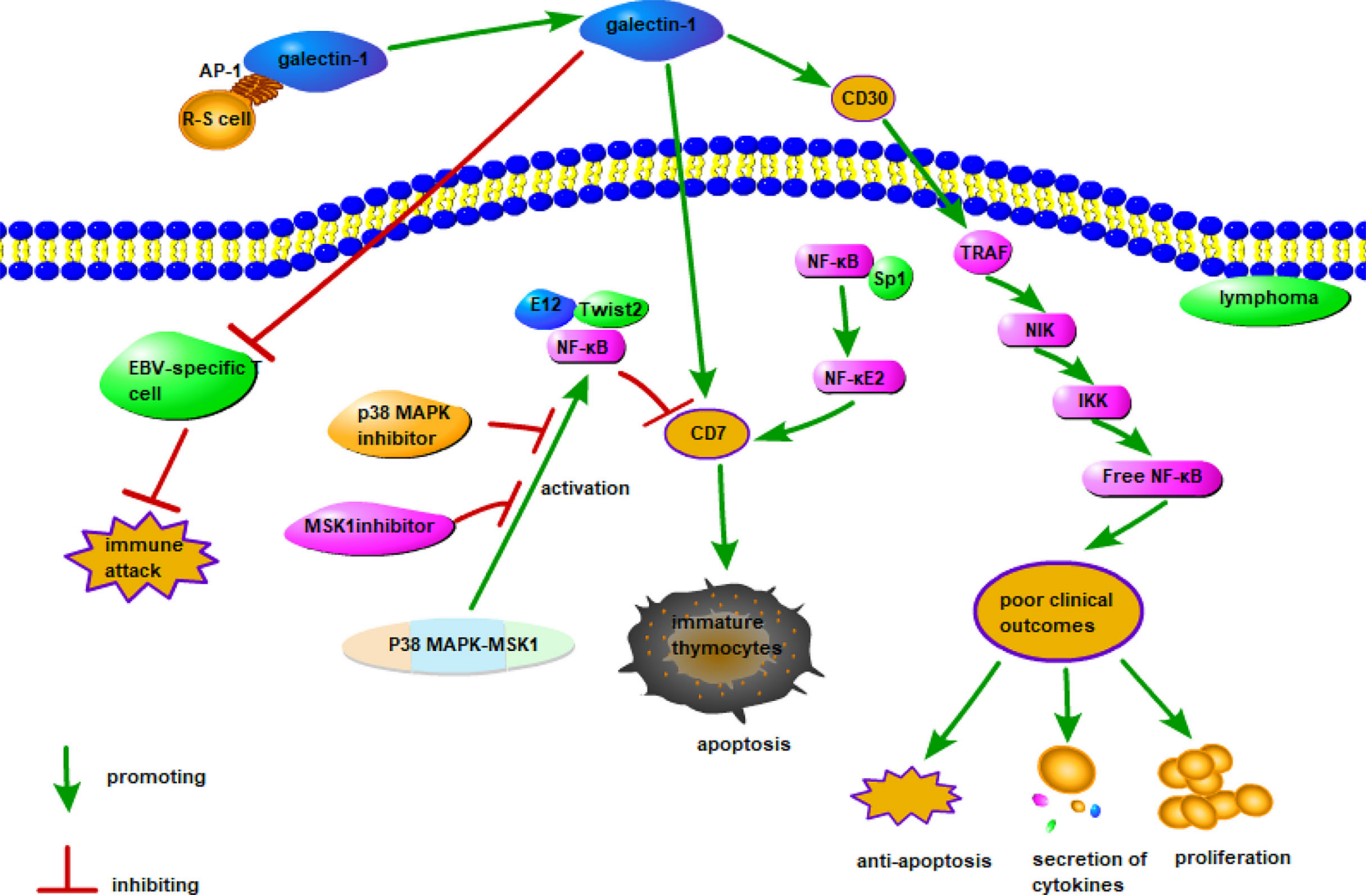
Possible mechanism of galectin-1 in lymphoma. The combination of AP-1 on the surface of R-S cells and galectin-1 promotes the expression of galectin-1, the combination of overexpressed galectin-1 and CD30 stimulates tumor necrosis factor–associated factor and activates the NF-κB signaling pathway to produce poor clinical outcomes. The combination of galectin-1 and CD7 induces apoptosis of immature thymocytes, the combination of NF-κB and Sp1 promotes the expression of CD7, while the combination of E12 and Twist2 with NF-κB inhibits the expression of CD7. Since the p38 MAPK-MSK1 pathway regulates CD7 expression by activating NF-κB, inhibitors of the p38 MAPK and MSK1 pathways can directly reduce CD7 expression. In addition, EBV-specific T cells binding to galectin-1 may inhibit immune attack.

Galectin-1 is overexpressed in lymphoma and plays important roles in this cancer. A possible mechanism of action of galectin-1 in lymphoma is shown in **Figure 1**. Galectin-1 is overexpressed in patients with classical Hodgkin’s lymphoma (cHL), particularly in Reed-Sternberg (R-S) cells (97). It is regulated by an activator protein-1 (AP-1)-dependent enhancer. This is a construct with a GC-rich regulatory element with an AP-1-binding site on R-S cells that selectively upregulates galectin-1 expression in cHL (96). Galectin-1 overexpression in R-S cells is a negative regulator of Epstein-Barr virus-specific T cell immunity and induces R-S cells to evade immune attack in cHL (98). It was also demonstrated that serum galectin-1 levels reflect the tumor burden and adverse clinical characteristics of cHL (99). Proteomics confirmed that galectin-1 expression in the tumor microenvironment is associated with poor clinical outcomes of cHL (100). Therefore, galectin-1 may be used as a prognostic biomarker for relapsed/refractory cHL (100).

Anaplastic large cell lymphoma (ALCL) overexpresses galectin-1, and the expression level is strongly correlated with c-Jun in the AP-1 transcription complex (101). Because most non-mediastinal diffuse large B-cell lymphomas (DLBCL) and mediastinal large B-cell lymphomas do not express galectin-1 and c-Jun, the combination of galectin-1 and c-Jun can be used as a diagnostic biomarker to distinguish other lymphomas with the same morphological or molecular characteristics as cHL and ALCL.

Two-thirds of cutaneous T cell lymphoma (CTCL) patients overexpress galectin-1, and this protein induces T cell apoptosis by binding to the T cell surface glycoprotein, CD7 (9). In CTCL, tumor-secreted galectin-1 inhibits the viability, proliferation, and Th1 response of non-malignant T cells and promotes the Th2 response that is conducive to tumor survival (102). Furthermore, galectin-1 is a key regulator of early CTCL keratinocyte proliferation (103). Therefore, inhibiting the secretion and expression of galectin-1 might be an effective strategy to delay the progression of CTCL (103).

A lack of CD7 expression in Sezary cells reduces their sensitivity to galectin-1-induced apoptosis and provides these cells with a survival advantage (104). It has been demonstrated that galectin-1 in the tumor microenvironment weakens the sensitivity of lymphomas to CD20 immunotherapy (105). The prognosis of peripheral T-cell lymphoma patients is significantly poor, and high intratumoral galectin-1 expression before treatment was associated with adverse outcomes in a cohort of patients with CD30^+^ and ALK^−^ peripheral T-cell lymphoma (106). HIV infection reduces the expression of highly soluble galectin-1, which leads to a pro-inflammatory but ineffective T cell response that ultimately promotes HIV-associated lymphoma. However, there are different opinions regarding the role of galectin-1. In HIV-associated DLBCL, patients with a higher intratumoral galectin-1 expression level have a higher survival rate (107). In addition, galectin-1 induced the death of ALCL cells, and this effect was more obvious when combined with CD30 pre-stimulation (108). Other studies have shown that galectin-1 promoted cell death by inhibiting the activity of CD45 protein tyrosine phosphatase (109). Although galectin-1 inhibitors and antibodies have been developed (**Table 2**), further studies are needed to explore their clinical effectiveness.

**Table 2:** The tumor-related clinical trials targeting on galectin family molecules.

Targets	Interventions	Disease	Phase	Status	Trail ID
Galectin-1	Biomarker analysis Pidilizumab	Stage III ~IV diffuse large B-cell lymphoma	II	Terminated	NCT02530125
Galectin-1	Brentuximab Vedotin Ipilimumab Nivolumab	Recurrent/Refractory classical Hodgkin’s Lymphoma	II	Recruiting	NCT01896999
Galectin-1	OTX008	Solid tumors	I	Unknown	NCT01724320
Galectin-3	GM-CT-01 5-Fluorouracil Leukovorin Bevacizumab	Colorectal cancer	II	Withdrawn	NCT00388700
Galectin-3	GM-CT-01 5-Fluorouracil	Cancer of the bile duct, Gallbladder cancer	II	Withdrawn	NCT00386516
Galectin-3	GM-CT-01 5-Fluorouracil	Colorectal cancer	II	Terminated	NCT00110721
Galectin3	GM-CT-01 5-Fluorouracil	Colorectal cancer, Lung cancer, Breast cancer, Head and neck cancer, Prostate cancer	I	Completed	NCT00054977
Galectin-3	Biomarker analysis	Cancer Survivor	II	Active, not recruiting	NCT01347970
Galectin-3	Blood sampling	Cancer, Leukemia, Hodgkin Lymphoma, Testicular cancer, Osteosarcoma, Ewing sarcoma, Breast cancer, Cervical cancer	–	Not yet recruiting	NCT05062707
Galectin-1,3	Sublingual videomicroscopy Blood sample	Von Willebrand diseases, Glanzmann thrombasthenia	Not Applicable	Not yet recruiting	NCT04119908
Galectin-3	GR-MD-02 Ipilimumab	Metastatic melanoma	I	Completed	NCT02117362
Galectin-3	Biomarker analysis	Thyroid cancer	–	Active, not recruiting	NCT03488134
Galectin-3	Biomarker analysis	Thyroid cancer, Papillary thyroid cancer, Follicular thyroid cancer	–	Recruiting	NCT04948437
Galectin-3	MAGE-3. A1 and/or NA17.A2 GM-CT-01	Metastatic melanoma	II	Terminated	NCT01723813
Galectin-3	GR-MD-02 Pembrolizumab	Melanoma, Non-small cell lung cancer Squamous cell carcinoma of the head and neck	I	Active, not recruiting	NCT02575404
Galectin-3	GR-MD-02 Placebo Pembrolizumab	Metastatic melanoma, Head and neck squamous cell carcinoma	II		NCT04987996
Galectin-3	Research Cardiac MRI Biomarkers	Breast cancer	–	Unknown	NCT02496260
Galectin-3	Research Cardiac MRI Biomarkers	Breast cancer	–	Completed	NCT02494453
Galectin-3	Biomarker analysis	Colon cancer, Rectal cancer	–	Completed	NCT01511653
Galectin-3	Biomarker analysis	Breast cancer	–	Unknown	NCT03155802
Galectin-3	Subclinical cardiac lesions and biomarkers	Breast cancer, Cardiac Toxicity	Not Applicable	Unknown	NCT02605512
Galectin-3	Cardiac imaging and circulating biomarkers	Breast cancer female	Not Applicable	Unknown	NCT03297346
Galectin-3	PectaSol-C Modified Citrus Pectin (MCP)	Prostatic neoplasms	II	Completed	NCT01681823
Galectin-9	Flow cytometric analysis	Gastrointestinal cancer	–	Completed	NCT04566848
Galectin-9	Flow cytometric analysis	Colorectal cancer	–	Recruiting	NCT04540159
Galectin-9	LYT-200 Anti-PD-1 Gemcitabine/nab-paclitaxel	Metastatic cancer, Solid tumor, Cholangiocarcinoma, Colorectal cancer, Pancreatic cancer	II	Recruiting	NCT04666688
Galectin-9	Tissue sampling Blood sampling	Cancer	Not Applicable	Recruiting	NCT04349293

## Galectin-3

The molecular weight of galectin-3 is 29-35 kDa, and it is encoded by the LGALS3 gene located on chromosome 14 (37). Galectin-3 is the only single chimeric protein of the galectin family, consisted of three structurally distinct domains: a short amino terminal, collagen-like structures, and a COOH-terminal CRD (C-CRD) containing the NWGR anti-death motif from the CRD and B-cell lymphoma-2 (Bcl-2) family (110). Galectin-3 is a multifunctional protein mainly located in the cytoplasm. It is shuttled between the cytoplasm and nucleus and transported to the cell membrane and extracellular environment through non-classical secretory pathways (110). In the cytoplasm, galectin-3 inhibits cell apoptosis by binding to ligands including Bcl-2, CD95, and Alix/AIP1 (111). In the nucleus, galectin-3 acts as a splicing factor for pre-mRNA and functions in spliceosome assembly (111). Galectin-3 in cell membranes and the extracellular matrix mediates cell adhesion, migration, and growth by binding with its ligands (laminin and fibronectin) (112).

**Figure 2:**
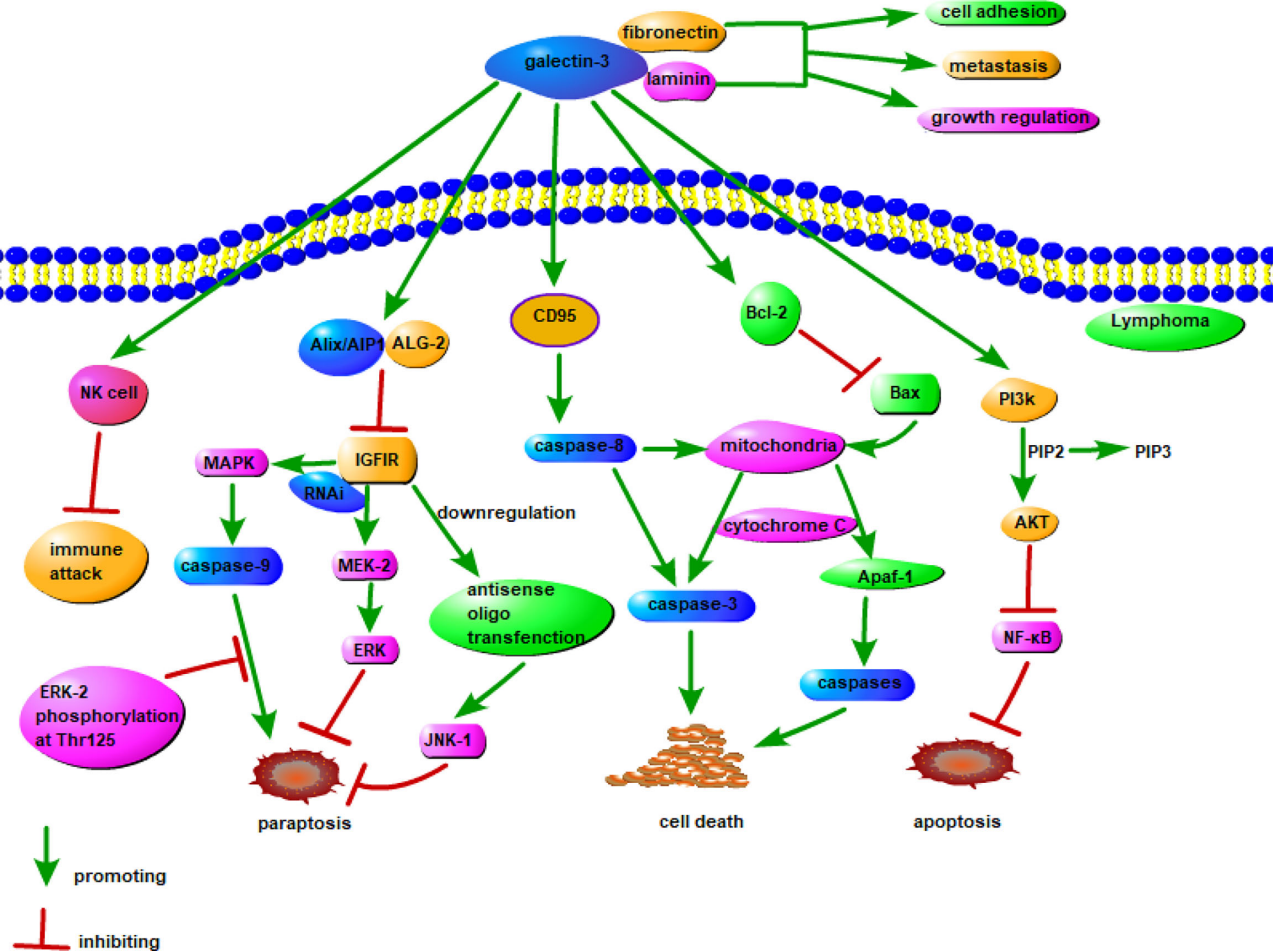
Possible mechanism of galectin-3 in lymphoma. Insulin-like growth factor I receptor (IGFIR) triggers the involvement of at least two signaling pathways, namely the MAPK/ERK and JNK pathways, leads to paraptosis. Both pharmacological inhibition of MAPK and downregulation of MEK-2 by RNAi, as well as downregulation of JNK1 by antisense oligo transfection, inhibites paraptosis. Among them, caspase-9 is a direct target of MAPK, and the phosphorylation of ERK-2 to Thr125 inhibits the pro-apoptotic activity of caspase-9. Galectin-3 inhibits IGFIR in combination with Alix/AIP1, thereby modulating paraptosis. The combination of galectin-3 and CD95 stimulates the activation of caspase-8 and interferes with the apoptotic signaling pathway from caspase-8 to mitochondria, and it can also combine with Bcl-2 to stimulate Bax and interfere with the apoptosis signaling pathway of mitochondrial Apaf-1. Galectin-3 can also inhibit apoptosis through PI3K/AKT/NF-κB signaling pathway. The combination of galectin-3 with NK cells may have the effect of suppressing immune attack, and the combination with fibronectin and laminin can promote tumorigenesis in lymphoma.

Galectin-3 is overexpressed in many tumors and positively correlates with the degree of tumor malignancy. It promotes the formation, progression, metastasis, and recurrence of tumors (110). Galectin-3 also suppresses tumor cell apoptosis *via* competing for a conserved structure with Bcl-2, suppressing cyclin, and increasing cell cycle inhibitors (113, 114). Galectin-3 regulates the phosphoinositide 3-kinase/Akt signaling pathway and enhances the activity of the anti-apoptotic factor, NF-κB (115). It stimulates early angiogenesis, accelerates the infiltration of tumor cells into the basement membrane and matrix, enhances vascular permeability, and promotes tumor cell extravasation (116). Galectin-3 also has important roles in tumor immunity. It interferes with the binding of natural killer cells to tumor cells, thereby evading the ability of natural killer cells to kill tumor cells (117). Extracellular galectin-3 binds to glycoproteins on the surface of T cells to induce T cell apoptosis (118). The possible mechanism of action of galectin-3 in lymphoma is shown in **Figure 2**.

Gene chip detection has demonstrated that galectin-3 is expressed in DLBCL patients but not in low-grade follicular lymphoma (FL) patients, providing one of the best means to distinguish DLBCL from FL (119). Histochemical staining confirmed the high expression levels of galectin-3 in DLBCL patients, and further research showed that galectin-3 protected B cells against Fas-induced apoptosis (120). Galectin-3 is also highly expressed in patients and cell lines of primary exudative lymphoma but not Burkitt’s lymphoma, marginal zone lymphoma, and small B-cell lymphoma (120). The expression level of galectin-3 is lowest in germinal center B cells and highest in primitive B cells (CD17^−^/IgD^+^) and memory B cells (CD10^−^/CD27^+^/IgD^−^) (120, 121). Galectin-3 combines with 90K to form a galectin-3/90K complex that promotes cell adhesion. It was demonstrated that high levels of 90K and galectin-3 were directly related to a poor response to therapy, high invasiveness, and short survival in patients with DLBCL (122).

A tissue chip assay was used to detect the expression of galectin-3 in 259 cases of primary DLBCL. The results showed that galectin-3 was localized to several subcellular sites and cell surfaces (34). In that study, after galectin-3 glycan inhibitor GCS-100 was used to remove galectin-3 from the surface of DLBCL cells, the cells were sensitive to apoptosis induced by dextran, rituximab, and etoposide (34). An immunoprecipitation assay confirmed that CD45 was the main counterreceptor of galectin-3 on the cell surface. In addition, removing galectin-3 from cell surface CD45 enhanced the phosphorylation activity, thereby increasing the sensitivity of DLBCL cells to chemotherapeutic drug-induced death. In contrast, galectin-3 can bind to specific O-glycans on CD45, reducing tyrosine phosphatase activity and thereby having anti-apoptotic effects in DLBCL (34). Additional studies found that the anti-apoptotic activity of galectin-3 in DLBCL mainly occurred on the cell surface. One study demonstrated that galectin-3 was overexpressed in all cases of Ki-1+ ALCL and might be a potential marker of this lymphoma (123).

Mitteldorf et al. compared the expression levels of galectin-3 in primary cutaneous anaplastic large cell lymphoma and lymphoid papulosis and found no difference, except for a different localization (35). The presence of endothelial hyperplasia and overexpression of galectin-3 in endothelial cells were considered prognostic factors for a poor primary central nervous system lymphoma outcome with normal immune function (124). Interestingly, the expression levels of galectin-3 in sera of non-Hodgkin’s lymphoma patients were related to cardiovascular events, and serum galectin-3 might be a prognostic biomarker for cumulative cardiovascular events (36).

Galectin-3 is widely expressed in stromal cells of adult T cells/lymphoma (ATLL) (125). Galectin-3 binding to CD7 induced tumor cell apoptosis, while lymphoma cells resisted exogenous galectin-3-induced apoptosis, resulting in a poor prognosis in ATLL (125). Therefore, galectin-3 may be used as an indicator of poor prognosis of lymphoma. Overall, research on the function of galectin-3 in lymphoma requires further exploration.

## Galectin-7

Galectin-7 has a molecular weight of 15 kDa and is encoded by the LGALS7 gene located on chromosome 19 (126). Galectin-7 is localized in the cytoplasm and nucleus and is secreted extracellularly *via* a non-classical secretion pathway (126). Galectin-7 has a high degree of tissue specificity, and its expression is mostly restricted to stratified epithelial cells (127). The expression of galectin-7 is regulated by a variety of transcription factors. In addition, the P53 gene induces the expression of galectin-7 in colorectal cancer (128).

Intracellular galectin-7 promotes cell apoptosis by increasing the activity of caspase-3 (129), accelerating the release of cytochrome C, and enhancing the activity of amino-terminal kinases that play important roles in maintaining epidermal homeostasis (129, 130). Galectin-7 is also involved in cell adhesion and migration and functions in wound healing, cancer progression, embryonic development, allergic inflammation, autoimmune diseases, and transplant rejection (131). Galectin-7 increases the expression levels of matrix metalloproteinase (MMP)-9, which has vital roles in tumorigenesis, metastasis, migration, and invasion *via* regulating extracellular signal-regulated kinase, c-Jun N-terminal kinase, and p38 mitogen activated protein kinase signaling pathways (132, 133).

Overexpression of galectin-7 inhibits the formation of new blood vessels, resulting in significant inhibition of the growth of colon cancer cells in mice (64). Galectin-7 acts similarly to galectin-1 in reducing the growth of neuroblastoma cells, without involving classical apoptosis, thereby playing a key role in spontaneous regression of neuroblastoma (54). DNA methylation induced galectin-7 and is usually related to the evolution of lymphoma cells into highly aggressive tumor cells (134). It was reported that high expression of galectin-7 in 164T2 lymphoma cells was associated with an increased recurrence rate and poor prognosis (55). Subsequent studies showed that the expression of galectin-7 was related to the DNA hypomethylation of its promoter (55).

**Figure 3:**
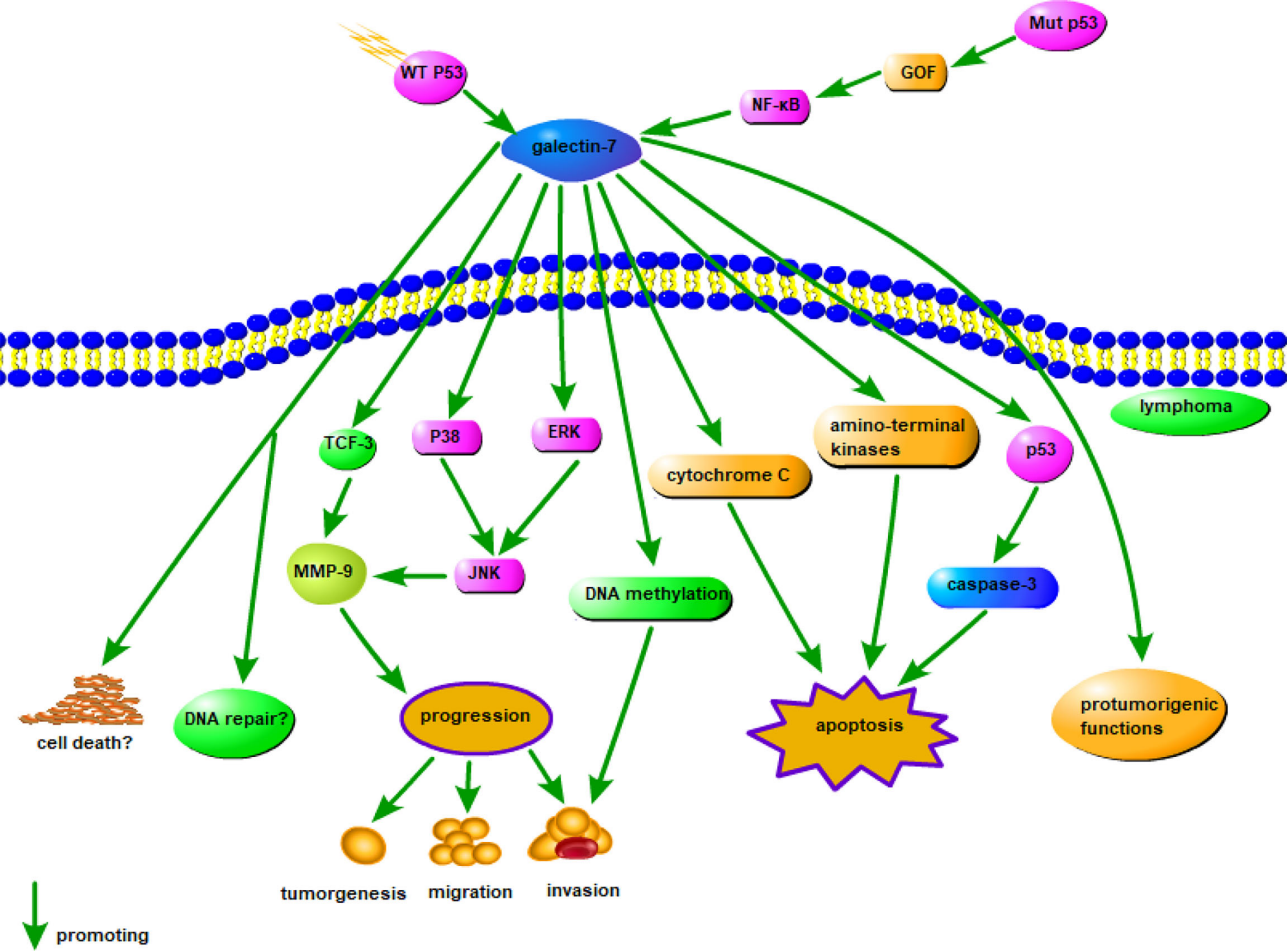
Possible mechanism of galectin-7 in lymphoma. MMP-9 overexpression is significantly related to the aggressive progression of lymphoma, and intracellular galectin-7 increases MMP-9 expression by TCF-3, while extracellular increases MMP-9 expression through P38, ERK, and JNK pathways. WT p53-induced galectin-7 expression induced by post-stress signaling can regulate cell death and/or DNA repair, and in cancer cells, galectin-7 can be induced by mutation p53 by a gain-of-function (GOF) mechanism, shifting balance to pro-tumor effects. In addition, DNA methylation, cytochrome C and amino-terminal kinases may also cause apoptosis by the action of galectin-7.

Galectin-7 accelerates the development of lymphoma cells and increases the metastatic behavior of low metastatic lymphoma cells *via* MMP-9 (135). The specific mechanism of action of galectin-7 in lymphoma has not been elucidated, but based on its general role in cancer, the mechanism is summarized in **Figure 3**. Galectin-7 is overexpressed in mature neoplastic B-cells rather than normal B cells (136). Galectin-7 cDNA transfection significantly suppresses the dissemination and invasion of lymphoma cells and increases the survival of mice. Inhibition of galectin-7 in aggressive lymphoma cells is related to reduced invasion by tumor cells and decreased expression of MMP-9 (136). Overall, the positive regulatory effect of galectin-7 on lymphoma provides us with a new therapeutic direction. Furthermore, the ability to inhibit galectin-7 to decrease tumor invasion and metastasis may become a new therapeutic strategy for lymphoma.

## Galectin-9

Galectin-9 has a molecular weight of 36 kDa and is encoded by the LGALS9 gene located on chromosome 17 (137). Galectin-9 was first isolated from mouse embryonic kidney tissue in 1997 and was cloned from the tumor tissue of nodular sclerosing Hodgkin’s lymphoma (137). Galectin-9 contains two different but homologous CRDs (N-CRD and C-CRD) that differ in inducing T cell death and activating dendritic cells. The C-CRD of galectin-9 mainly determines receptor recognition and T cell death pathway signaling, while the N-CRD mainly activates dendritic cells (138). Previous studies have shown that galectin-9 is widely distributed in the liver, spleen, stomach, colon, lymph nodes, appendix, gallbladder, bone marrow, lung, and bladder and various cells, including eosinophils, epithelial cells, endothelial cells, T lymphocytes, dendritic cells, and macrophages (137).

Intra- and extracellular galectin-9 interacts with ligands to regulate biological functions. A variety of galectin-9 surface-binding ligands have been reported, such as T cell immunoglobulin mucin-3 (Tim-3), cell surface protein disulfide isomerase, CD44, 4-1BB (CD137), glucose transport protein-2, Forssman glycosphingolipid, IgE, and IgM (137, 139). When combined with its ligands, galectin-9 is implicated in the occurrence and development of various autoimmune diseases, transplant rejection, allergic diseases, infections, and tumors (137). The most characteristic ligand of galectin is Tim-3. This ligand is widely expressed on the surface of immune cells and induces Th1 and Th17 cell apoptosis after binding with galectin-9 (140). Activating the galectin-9/Tim-3 pathway suppresses the immune response by inducing the proliferation of bone marrow-derived suppressive cells and leads to the failure of T cells (141, 142). Moreover, Tim-3 plays important roles in the process of anti-programmed cell death 1 (PD1)/programmed cell death ligand 1 (PD-L1) treatment resistance (143). The galectin-9/Tim-3 signaling pathway was shown to be a key mechanism of resistance to anti-PD1 immunotherapy (77). Therefore, galectin-9/Tim-3 inhibitors may be an effective treatment to enhance the efficacy of PD1/PD-L1 antibodies.

**Figure 4:**
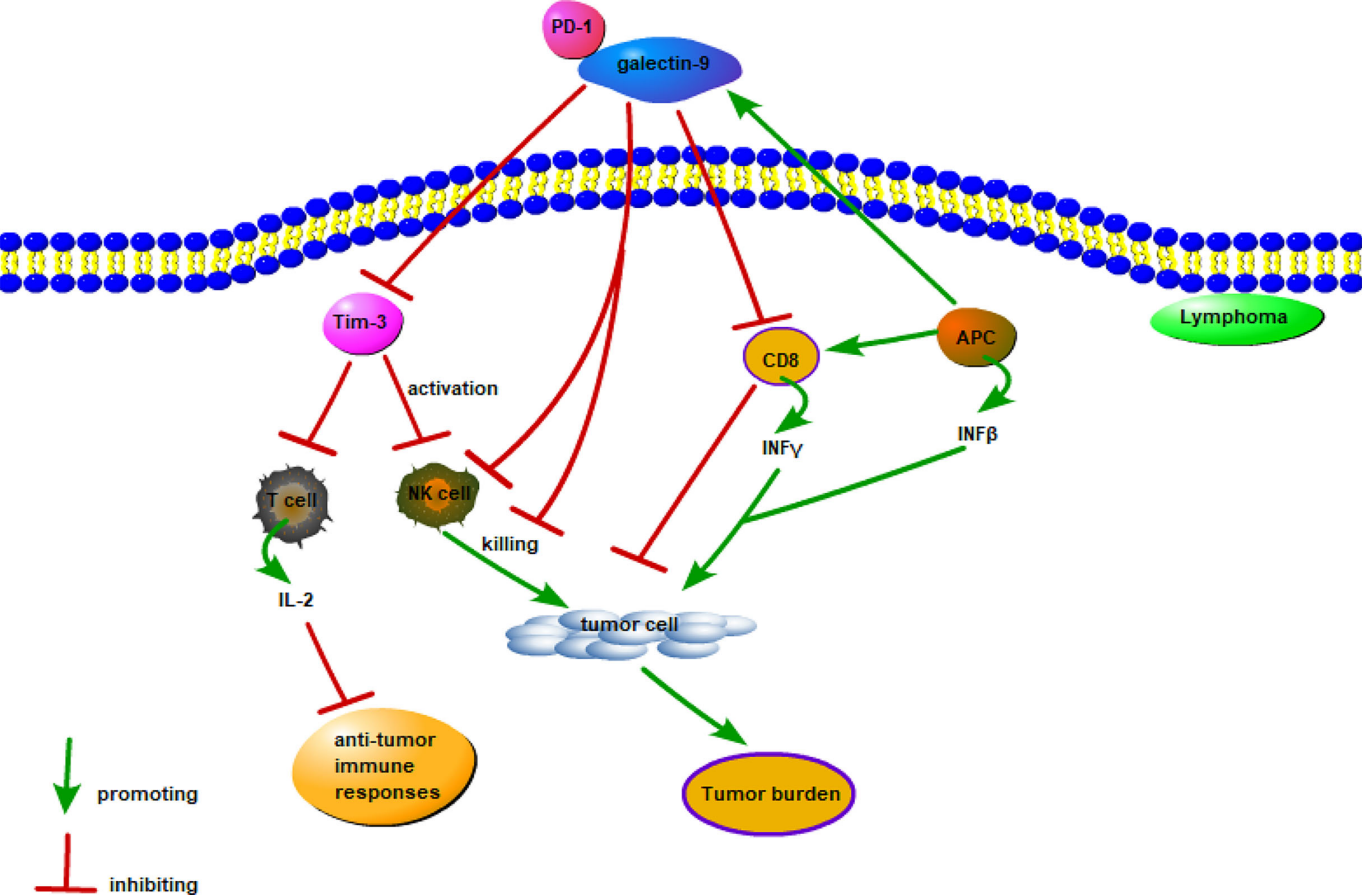
Possible mechanism of galectin-9 in lymphoma. Galectin-9 mainly exerts a pro-tumor effect by binding to Tim-3, Tim-3 inhibits the cytotoxic IL-2 secreted by T cells and inhibits the lethality of NK cells, and the combination of PD-1 and galectin-9 weakens the work of Gal-9/Tim-3. Interferon-induced expression and secretion of galectin-9 is a potential mechanism for tumor-acquired immune resistance. INFβ produced by APC and tumor cells and INFγ produced by activated CD8 T cells induce APC and tumor cells to express and secrete galectin-9. However, galcectin-9 induces T cell death and inhibits the anti-tumor immune response.

The expression of galectin-9 is far less extensive than that of galectin-1 and galectin-3 in lymphoma. Primarily, galectin-9 is increased in patients with various infectious diseases and allergies (144). The possible mechanism of galectin-9 in lymphoma is shown in **Figure 4**. In ATL/ATLL, increased plasma galectin-9 level indicates the tumor burden and reflects opportunistic infections resembling the immune reconstitution inflammatory syndrome due to mogamulizumab therapy (144). Therefore, increased galectin-9 level might reflect immune-related adverse effects of lymphoma biotherapy (144).

Galectin-9 is overexpressed on tumor cells in lesional skin of CTCL (7). The expression levels correlate with reduced CD8^+^ T-cell infiltration and disease severity markers (7). Galectin-9 promotes CTCL cell death *via* activating caspase-3 and caspase-9, which elicits apoptosis and inhibits the growth of CTCL cells (7). An anti-Tim-3 blocking antibody combined with galectin-9 strengthens the suppression of CTCL growth (7). Galectin-9/Tim-3 co-blockade has been studied extensively in other tumors (143) and may be developed as a new therapy against PD1/PD-L1-resistant lymphoma.

## Conclusion

In summary, the widespread expression of galectin family proteins in tissues is inseparable from the occurrence, development, invasion, and metastasis of tumors. Importantly, the different galectin expression levels in normal and tumor tissues create the possibility of this family functioning as biomarkers for detecting cancer progression and serving as targets for improving the clinical prognosis. Overall, using galectin as a novel target provides new approaches for improving the diagnosis, treatment, and prognosis of lymphoma. Preclinical experiments have shown that inhibiting galectins effectively decreases tumor progression. However, the clinical exploration of galectin inhibitors is still in the preliminary stage, and whether they can be used in cancer treatment requires further research. Nevertheless, in recent decades, research into the roles of galectins in tumors has made significant progress and led to a number of galectin inhibitors entering clinical trials. Clinical studies investigating the use of galectin inhibitors in tumors, recognized by the National Institutes of Health (https://clinicaltrials.gov/), are shown in **Table 2**. These clinical studies mainly focus on the detection of biomarkers and the application of galectin inhibitors and monoclonal antibodies. However, due to the lack of clinical trials of galectin inhibitors, the efficacy and side effects of galectin inhibitors in the human body have not been systematically elucidated, so the clinical application of galectin inhibitors is challenging. In the future, further researches are needed on the role and mechanism of galectins in lymphoma and tumors, so as to provide new solutions for the treatment of lymphoma and other cancers.

## Author Contributions

All authors participated in the development, writing, and editing of the review article. All authors contributed to the article and approved the submitted version.

## Funding

This work was supported by grants from the key research project program of Shandong Province (2018GSF118035), the Medical Health Science and Technology Development Plan of Shandong Province (2017–462), the Affiliated Hospital Development Fund of Xuzhou Medical University (XYFM2020016) and Zhejiang Provincial Natural Science Foundation of China (LZ22H030003).

## Conflict of Interest

The authors declare that the research was conducted in the absence of any commercial or financial relationships that could be construed as a potential conflict of interest.

## Publisher’s Note

All claims expressed in this article are solely those of the authors and do not necessarily represent those of their affiliated organizations, or those of the publisher, the editors and the reviewers. Any product that may be evaluated in this article, or claim that may be made by its manufacturer, is not guaranteed or endorsed by the publisher.

## Abbreviations

ALCL, anaplastic large cell lymphoma; AP-1, activator protein-1; ATL/ATLL, adult T-cell leukemia/adult T cells lymphoma; Bcl-2, B-cell lymphoma-2; cHL, classical Hodgkin’s Lymphoma; c-Jun, a transcriptional regulator of leucine zipper family; CRD, carbohydrate recognition domains; CTCL, cutaneous T cell lymphoma; DLBCL, diffuse large B-cell lymphoma; FL, follicular lymphoma; NF-κB, nuclear factor kappa-B; MMP, matrix metalloproteinase; PI3K/Akt, a proto-oncogene and a signaling pathway related to phosphatidylinositol; R-S cells, Reed-Sternberg cells; Tim-3,T cell immunoglobulin mucin-3.
